# Flubendazole Plays an Important Anti-Tumor Role in Different Types of Cancers

**DOI:** 10.3390/ijms23010519

**Published:** 2022-01-04

**Authors:** Chaoran Chen, Yueming Ding, Huiyang Liu, Mengyao Sun, Honggang Wang, Dongdong Wu

**Affiliations:** 1 Institute of Nursing and Health, School of Nursing and Health, Henan University, Jinming Avenue, Kaifeng 475004, China; kfccr@henu.edu.cn (C.C.); dym15375320339@163.com (Y.D.); 2 Henan International Joint Laboratory of Nuclear Protein Regulation, School of Basic Medical Sciences, Henan University, Kaifeng 475004, China; m15736875597@163.com; 3 School of Clinical Medicine, Henan University, Kaifeng 475004, China; smy15290790500@163.com; 4 School of Stomatology, Henan University, Kaifeng 475004, China

**Keywords:** flubendazole, anti-tumor role, breast cancer, melanoma, prostate cancer

## Abstract

Flubendazole, belonging to benzimidazole, is a broad-spectrum insect repellent and has been repurposed as a promising anticancer drug. In recent years, many studies have shown that flubendazole plays an anti-tumor role in different types of cancers, including breast cancer, melanoma, prostate cancer, colorectal cancer, and lung cancer. Although the anti-tumor mechanism of flubendazole has been studied, it has not been fully understood. In this review, we summarized the recent studies regarding the anti-tumor effects of flubendazole in different types of cancers and analyzed the related mechanisms, in order to provide the theoretical reference for further studies in the future.

## 1. Introduction

**Figure 1:**
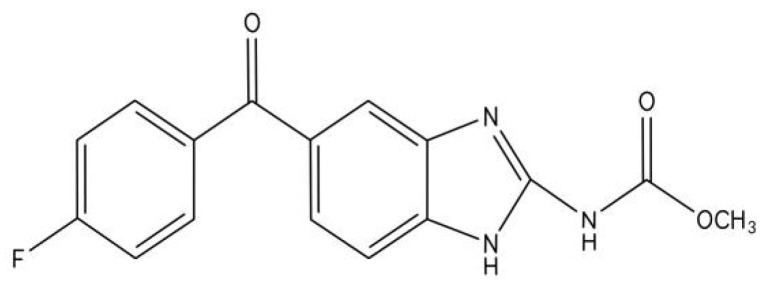
The structure of flubendazole.

Flubendazole(FLU)([5-(4-fluorobenzoyl)-1H-benzimidazole-2-y1]-carbamic acid methyl ester) (Figure 1), which was developed by Janssen in the 1970s, belongs to the group of benzimidazole anthelmintics and is widely used in the treatment of helminth and intestinal parasite infection [1,2,3,4,5]. It has a typical benzimidazole part, but the main structure is the added fluorine atom, which makes it different from other benzimidazoles [6]. In 1980, FLU was approved for the treatment of gastrointestinal nematode infection in veterinary and human medicine. It has also been known for a long time that the intravenous FLU can lead to a high level of filarial effect in many preclinical models and humans [7]. The anthelmintic effect of FLU is based on its ability to change microtubule structure, inhibit tubulin polymerization, destroy microtubule function, and interfere with the transport of secretory vesicles mediated by the microtubule in the absorptive tissues of helminths [4,8,9,10,11]. In recent years, FLU has been found to activate autophagy and to be an effective inducer of reactive oxygen species (ROS) [12,13]. It has been reported that FLU plays an important anti-tumor role in many kinds of cancers, such as breast cancer, melanoma, neuroblastoma, colorectal cancer, liver cancer, oral squamous cell cancer [1,10,14,15,16,17,18,19,20,21,22,23,24], and is regarded as a promising anti-tumor drug [1,22]. Recent studies have shown that FLU inhibits cell proliferation and delays tumor formation in xenograft models and shows preclinical activity by suppressing tubulin polymerization in lymphoma and leukemia [10,25,26]. Although some studies have described some anti-tumor mechanisms of FLU, including the inhibition of the tumor cell cycle, and the tumor cell proliferation and growth, the anti-tumor mechanism of FLU has not been fully understood [10,22,23,27]. Here, we reviewed and analyzed the studies about the anti-tumor effects and mechanisms of FLU in recent years, hoping to provide a theoretical reference for further studies on the anti-tumor effect of FLU in the future.

## 2. The Pharmacokinetics of Flubendazole

FLU is usually taken orally and absorbed through the gastrointestinal tract. Only a small amount of FLU is reabsorbed after the oral treatment in pigs, rats, sheep, dogs, and humans [28]. FLU is difficult to dissolve in the water system of the gastrointestinal tract, resulting in the low absorption rate of blood, so its bioavailability is very low. Even if the oral dose in humans was 2 g, the maximum plasma concentration level of FLU in humans was lower than 5 ng/mL. If FLU is used immediately after meals, its absorption increases significantly [20,29]. In order to ameliorate the bioavailability of the system, it has been reconstituted as an amorphous solid dispersion (ASD) oral preparation [3]. After subcutaneous administration, FLU showed high effectiveness in the animal model of filarial infection. FLU has obvious clinical effects on onchocerciasis by intramuscular injection in humans. However, the intramuscular injection leads to severe injection site reactions [30].

There are two possible biotransformation ways of FLU in vivo. The first metabolic pathway is the transformation of absorbed FLU in the first-pass metabolic process of the liver, in which carbamate hydrolysis or ketone reduction may occur in the liver. The reduction of ketones to methylcarbamate is the main metabolic pathway in chickens and turkeys. The second metabolic pathway is the hydrolysis of carbamate to ketone, which is the main metabolic pathway in pigs. The products of the above two pathways are then converted to 2-amino-α-(4-fluorophenyl)-1H-benzimidazole-5-methanol [4,21,31].

More than 80% of the oral dose of FLU is excreted through feces, only small amounts of unchanged drugs (less than 0.1%) are excreted in the urine, and its half-life in tissues is 1–2 days [4].

## 3. The Mechanism of Action of Flubendazole

Microtubules are the cytoskeletal components that are required for cell division, cell transport, and maintaining cell integrity [32]. Microtubules consist of α- and β-tubulin heterodimers, which are assembled into the linear filaments and polymerized into the hollow fibers and cylindrical structures [33]. The acquisition or loss of tubulin heterodimers leads to the microtubules elongation or microtubules shortening [34]. The action mechanism of FLU consists of the possible binding of the tubulin specifically to change the microtubule structure, inhibiting the tubulin polymerization, and destroying the microtubule function, and then interfering with the microtubule-mediated physiological activities [4]. Due to the crucial role of the microtubule structure in many important functions of parasites, such as proliferation, mitosis, intracellular transport of organelles, maintenance of cell shape or cell movement, the changes in the microtubule assembly and dynamics leads to the final destruction of parasites [9,32]. In addition, FLU also inhibits the energy metabolism of parasitic cells. It can disrupt glucose transport and metabolism, resulting in energy depletion, reduction in glycogen reserves, and loss of cell viability. Thus, this process eventually leads to the death of parasites [35,36]. Microtubules are also involved in the invasion and metastasis of tumor cells by playing an important role in the proliferation, migration, and transport of eukaryotic cells [37,38].

## 4. Anti-Tumor Role of Flubendazole in Breast Cancer

### 4.1. Breast CS-like Cells Inhibition via Suppressing Cellcycle Progression and Tubulin Polymerization 

Breast cancer is the most common cancer in women and is also a common cause of cancer-related deaths [39,40,41]. Although the traditional therapies including surgery, radiotherapy, chemotherapy, and hormone therapy have improved the survival rate of patients with breast cancer, recurrence and metastasis are still unavoidable [42,43,44]. Cancer stem-like cells (CS-like cells) are considered to be the main cause of breast cancer recurrence and drug resistance, which makes it a potential target for the new cancer treatment strategies [45,46,47]. It has been reported that breast CS-like cells are enriched by chemotherapy and radiotherapy, indicating that CS-like cells have therapy resistance [48,49]. The results of Zhi-Jie Hou et al. showed that FLU suppressed the proliferation of breast cancer which enriched CS-like cells in a dose- and time-dependent manner and reduced the tumor volume of the xenograft model by intraperitoneal injection. The mechanism study revealed that FLU decreased CD44^high^/CD24^low^ subpopulation, inhibited mammosphere formation, and reduced the expression of self-renewal-related genes such as *c-Myc*, *Oct4*, *Sox2*, *Nanog* and *CyclinD1* in breast cancer cells, thus significantly reducing the properties of CS-like cells in breast cancer. Moreover, FLU also decreased the number of CS-like cells by inducing their differentiation. Collectively, it indicated that FLU suppressed breast cancer by reducing the properties and the number of CS-like cells in CS-like cells-enriched breast cancer. In addition, FLU suppressed the cell migration and reversed the epithelial-mesenchymal transition (EMT) phenotype by decreasing the expression of mesenchymal markers (β-catenin, N-cadherin, and Vimentin) and promoting the expression of the epithelial and differentiation marker (Keratin 18) in CS-like cells-enriched breast cancer, indicating that FLU inhibited breast cancer metastasis. The further study showed that FLU inhibited cell cycle at the G2/M phase and promoted the monopolar spindle formation by suppressing tubulin polymerization in breast cancer cells, which inhibited CS-like cells capability. FLU treatment significantly enhanced the cytotoxicity of fluorouracil and adriamycin on CS-like cells-enriched breast cancer and their inhibitory effect on the colony-forming ability of breast cancer cells, indicating that FLU could reduce the drug resistance of breast cancer. From the above, FLU could ameliorate breast cancer through inhibiting proliferation, metastasis, and drug resistance mainly by inhibiting breast CS-like cells in CS-like cells-enriched breast cancer via inhibiting cell cycle progression and tubulin polymerization [24].

### 4.2. Breast CS-like Cells Inhibition via Suppressing STAT3 Activation

In addition to the above mentioned that FLU suppressed breast CS-like cells proliferation by inhibiting the cell cycle at the G2/M phase and tubulin polymerization, the signal transducer and activator of transcription 3 (STAT3) signaling pathway is also involved in the inhibition of breast CS-like cells by FLU [23]. Three negative breast cancer (TNBC) is a particularly aggressive subtype of breast cancer with the negative expression of human epidermal growth factor receptor-2 (HER2), progesterone (PR), and estrogen (ER) [50,51,52]. The treatment of TNBC is challenging because of the lack of established molecular targets. At present, chemotherapy is still the main treatment for early and late TNBC patients [53,54,55]. The results of Oh et al. showed that FLU treatment could notably induce TNBC cells apoptosis and accumulation in the G2/M phase, and caspase-3/-7 activation and STAT3 inhibition in TNBC cells. Pretreatment with Z-VAD-fmk, the caspase inhibitor, completely inhibited FLU-induced apoptosis and caspase-3/-7, indicating that caspase-3/-7 mediated FLU-induced apoptosis. FLU impaired the properties of breast CS-like cells by decreasing CD24^high^/CD49f^high^, CD24^low^/CD44^high^ subpopulation, mammosphere formation, and ALDH1 activity. FLU also reduced STAT3 phosphorylation and cyclin D1 expression in the mammosphere-forming cells. S3I-201 and LLL12, two pharmacological inhibitors of STAT3 phosphorylation, had the synergistic inhibitory effects with FLU on mammosphere formation, while interleukin-6(IL-6), a STAT3 activator, had the opposite effects, indicating that FLU suppressed the properties of breast CS-like cells by inhibiting STAT3 pathway. FLU administration also inhibited breast CS-like cells-enriched TNBC tumor growth, angiogenesis, and metastasis to lung and liver through suppressing STAT3 activation. Collectively, FLU could improve TNBC mainly by targeting breast CS-like cells through inhibiting STAT3 activation [23]. The mechanism of FLU inhibiting breast CS-like cells has not been fully understood and needs to be further studied. Traditional cancer treatment kills most of the proliferating cancer cells that make up the tumor volume but often cannot eliminate CS-like cells, which increases the chance of tumor recurrence [56,57]. Therefore, it can be concluded that the inhibitory effect of FLU on CS-like cells is of great importance and could be a leading new approach to breast cancer treatment.

### 4.3. Improvement of Drug Resistance by Inhibiting CS-like Cells Properties and HER2 Pathway, and Inducing Apoptosis and G2/M Phase Arrest in Breast Cancer

Human epidermal growth factor receptor-positive (HER2-positive) breast cancer accounts for about 15–20% of breast cancers. HER2 is associated with aggressive tumor behavior and poor prognosis [58,59,60]. Although trastuzumab has significant clinical effects on HER2-positive breast cancer, the emergence of drug resistance limits the efficacy [61,62]. The main potential mechanism of anti-trastuzumab involves the interaction between HER2 and other family members, such as EGFR and HER3 [63]. The HER2 pathway is also involved in trastuzumab resistance [64,65,66]. The results of Yoon-Jae Kim et al. showed that FLU promoted G2/M phase arrest and decreased cell viability in HER2-positive breast cancer cells in vitro. In HER2-positive breast cancer, FLU also induced caspase-dependent apoptosis by activating caspase-3, caspase-7, and caspase-8, and suppressed trastuzumab-resistant cells proliferation through decreasing cell viability and inducing apoptosis and G2/M phase arrest. The mechanism study revealed that FLU inhibited the HER2 pathway in both trastuzumab-sensitive and trastuzumab-resistant HER2-positive breast cancer cells by decreasing the levels of the truncated p95HER2, phospho-HER2, phospho HER3, and phospho-Akt, and preventing the hetero-dimerization of HER2/HER3. Moreover, FLU inhibited HER2-positive breast CS-like cells properties by suppressing ALDH1 activity, CD44^high^/CD24^low^ phenotype, and mammosphere formation. FLU notably suppressed the tumor growth of trastuzumab-resistant xenografts by promoting apoptosis and downregulating the expression of p95HER2, ALDH1A1, and CD44 in vivo. Collectively, FLU surmounted the resistance to trastuzumab by inhibiting CS-like cells properties and HER2 pathway and induced apoptosis and G2/M phase arrest in HER2-positive breast cancer [22]. The effect of FLU on the HER2 pathway needs further study.

### 4.4. Promotion of Autophagic Cell Death of TNBC Cells through Upregulating EVA1A

It has been reported that FLU can also inhibit breast cancer cells proliferation by promoting autophagic cell death by significantly increasing ROS generation [12,13]. Eva-1 homolog A (EVA1A) is a lysosome and endoplasmic reticulum-associated protein. It regulates autophagy and apoptosis [67,68,69]. Yongqi Zhen and colleagues demonstrated that after treatment with FLU, the proliferation of TNBC cells was significantly inhibited and colony formation was reduced in vitro. Similar results were obtained in a mouse xenograft tumor model in vivo. The in-depth study showed that FLU increased the apoptosis of TNBC cells by upregulating the expression levels of bax and cleaved caspase 3 and downregulating bcl-2 expression. FLU also induced autophagy by increasing the levels of LC3-II/LC3-I and Beclin-1 and inducing p62 degradation. In addition, FLU could facilitate the formation of autolysosomes and autophagosomes. 3-MA (an autophagy inhibitor that inhibits autophagosome formation) inhibited autophagy and partially abolished TNBC cells apoptosis induced by FLU, indicating that FLU induced apoptosis of TNBC cells by promoting autophagy. FLU suppressed TNBC metastasis by increasing E-cadherin expression and reducing MMP-2 expression in vivo and in vitro, which was mitigated by the combined FLU treatment with 3-MA or ATG5 knockdown, indicating that FLU inhibited TNBC metastasis by promoting autophagy via ATG5. The expression of EVA1A was decreased in tumor tissues of TNBC, which was reversed by FLU in vivo and in vitro. EVA1A knockdown with siRNA mitigated the suppression of autophagy, metastasis, and apoptosis of TNBC cells by FLU, indicating that FLU induced autophagy and suppressed the proliferation and migration of TNBC cells through upregulating EVA1A. Furthermore, EVA1A overexpression also significantly inhibited TNBC cells’ growth and proliferation, which was alleviated in ATG5-depleted TNBC cells. Moreover, EVA1A overexpression could not induce LC3 aggregation in ATG5 knockdown TNBC cells, demonstrating that EVA1A-induced autophagy is dependent on ATG5. The above results indicated that FLU suppressed TNBC apoptosis and metastasis by inducing autophagy via ATG5. The studies on the combination of FLU and EVA1A revealed that the point mutation of Thr113 in EVA1A alleviated the suppression of autophagy, apoptosis, and metastasis of TNBC by FLU, indicating that FLU might promote EVA1A expression through binding Thr113 in EVA1A. Collectively, FLU could promote autophagic cell death of TNBC cells by promoting EVA1A, thus suppressing tumor proliferation and migration [70]. From the above, ATG5 mediated the EVA1A-regulated autophagic cell death of TNBC cells promoted by FLU. Furthermore, FLU has been reported to promote autophagy through ATG4B [13], therefore, whether ATG4B mediated the EVA1A-regulated autophagic cell death of TNBC cells promoted by FLU needs to be studied.

## 5. Anti-Tumor Role of Flubendazole in Melanoma

### 5.1. Suppression of Mitosis and Induction of Apoptosis in Melanoma Cells

Melanoma, an invasive cancer caused by melanocytes, not only locally invades the surrounding tissues but also metastasizes throughout the body [71,72,73]. It is a leading cause of cancer-related death [74]. It has been reported that FLU delays tumor growth by inhibiting the microtubule function in myeloma xenografts with no obvious cytotoxicity [10]. Čáňová K. and colleagues found that FLU suppressed cell growth and proliferation in malignant melanoma cell lines including A-375, BOWES, and RPMI-7951 cell lines. The mechanism study revealed that FLU induced melanoma cells accumulation at the G2/M phase of the cell cycle and destroyed the structure and function of microtubules, accompanied by the great changes in cell morphology. Moreover, FLU could cause abnormal mitosis, which is characterized by the formation of multinucleated giant cells and abnormal spindles. Lastly, FLU induced mitochondria and caspase-3/7-dependent apoptosis. Collectively, FLU suppressed melanoma by inhibiting mitosis and induced apoptosis in melanoma cells [14]. How FLU-induced abnormal mitosis caused mitochondria-mediated apoptosis remains to be clarified. p53 is a tumor suppressor gene, and its mutation is related to more than half of human tumors [75,76]. In the above studies, the effects of FLU on p53 are diverse in different melanoma cell lines, and its mechanism needs to be further studied.

### 5.2. Silence of the Immunosuppressive Effects of PD-1 and MDSC in Melanoma Cells

A study by Yue Li et al. showed that FLU could inhibit the growth and metastasis of melanoma when administered systemically rather than locally. FLU inhibited tumor angiogenesis—as evidenced by almost completely suppressing the level of CD31 and STAT3. The in-depth study revealed that FLU inhibited the expression of programmed death-1 (PD-1), not programmed death-ligand 1 (PD-L1), in cultured melanoma cells. Moreover, FLU also decreased the level of myeloid-derived suppressor cells (MDSC) and STAT3 in tumor tissues [20]. PD-1 and MDSC are negative regulators of the immune system [77,78]. STAT3 is an upstream regulator of PD-1 expression [79]. Therefore, these studies showed, for the first time, that FLU inhibited the growth and metastasis of melanoma by silencing the immunosuppressive effects of PD-1 and MDSC [20]. STAT3 regulates MDSC in head and neck tumors [80]. Therefore, the STAT3 pathway may mediate the FLU inhibition of PD-1 and MDSC in melanoma, which is further proven by using the STAT3 inhibitor.

## 6. Flubendazole Inhibits Prostate Cancer by Promoting P53 Signaling Pathway to Induce Ferroptosis and Cell Cycle Arrest

Prostate cancer (PCa) is the most common malignant tumor in men and the common cause of cancer-related deaths in western countries [81,82,83]. The current treatment for prostate cancer is androgen deprivation therapy (ADT). However, most patients treated with ADT develop castration-resistant human prostate cancer (CRPC) after 2–3 years. At present, there is no effective treatment for CRPC, therefore, it is particularly important to find an effective method to treat CRPC [84,85,86]. The results of Xumin Zhou et al. demonstrated that FLU inhibited the proliferation of CRPC cells in a dose- and time-dependent manner through upregulating P53 to induce cell cycle arrest in the G2/M phase. FLU also accelerated ferroptosis-induced cell death in CRPC cells by inhibiting ferroptosis-related contents, SLC7A11 and GPX4. The in-depth study showed that FLU improved the stability of p53 by binding to p53, indicating that p53 is the target gene of FLU. p53 can also bind to the promoter of SLC7A11. p53 depletion with siRNA upregulated SLC7A11 expression in CRPC cells, while p53 overexpression had the opposite effect. The above indicated that p53 regulated SLC7A11 by binding to its promoter. Therefore, it indicated that FLU promoted ferroptosis through upregulating P53 in CRPC cells. Moreover, FLU and 5-fluorouracil (5-FU) have synergistic effects in CRPC chemotherapy. p53 overexpression could enhance the synergistic effects of FLU and 5-FU, while p53 inhibition had the opposite effect, indicating that p53 mediated the synergistic effects of FLU and 5-FU in CRPC cells. In the in vivo xenograft models, FLU notably increased p53 expression and promoted ferroptosis, which was consistent with the experimental results in vitro. Overall, FLU suppressed CRPC growth by promoting the p53 signaling pathway to induce ferroptosis and cell cycle arrest [87]. In addition to the p53 signaling pathway, it has been reported that FLU inhibits tumor via NF-κB pathway and STAT3 pathway [1,15,23], therefore, whether the above two pathways are involved in the suppression of CRPC by FLU needs to be studied. From the above studies, we know that FLU has an inhibitory effect on CRPC cells at low concentrations. Therefore, the combination of FLU and other drugs in the treatment of CRPC should be the focus of future research.

## 7. Flubendazole Inhibits Lung Cancer Proliferation by Promoting Autophagic Cell Death

Lung cancer is the most common cause of cancer-related deaths in the world. It is estimated that 1.6 million people die from lung cancer every year. About 85% of lung cancer is non-small cell lung cancer (NSCLC) [88,89,90]. Tingjun Dong and colleagues found that FLU was a potential chemotherapeutic drug for lung cancer because it could inhibit the proliferation of NSCLC cells and reduce the viability of NSCLC A549 and H460 cells. A further study showed that FLU could promote autophagy by upregulating the ratio of LC3 II/I, reducing p62 protein, and promoting the activation of autophagy flow in A549 and H460 cells [27]. Whether FLU could suppress the proliferation of NSCLC cells through inducing autophagic cell death needs to be further elucidated by using an autophagy inhibitor. Moreover, the involved signaling pathways need to be determined.

## 8. Flubendazole Inhibits Colon Cancer by Suppressing Mitosis and Inducing Apoptosis and Cell Senescence

Colon cancer is the second leading digestive cancer worldwide. The recurrence and chemoresistance are very common in advanced colon cancer. Therefore, it is urgent to find effective drugs to treat colon cancer [91,92,93]. It has been reported that FLU significantly inhibited cell proliferation in a concentration-dependent and time-dependent manner by arresting the G2/M phase [25]. Vera Krlova and colleagues found that FLU significantly inhibited the proliferation of colon cancer cells. The mechanism study showed that FLU decreased the level of cyclin D1, increased the level of cyclin B1, activated caspase 2, caspase 3/7, and cleaved PARP. The morphological observation showed that the microtubule network was destroyed, with the irregular mitotic spindle, the formation of huge multinucleated cells, and the increase of nuclear area and DNA content. The above indicated that FLU induced apoptosis and mitotic arrest in colon cancer cells. FLU also induced senescence as evidenced by the increase in SA-β-galactosidase (an aging-related indicator) level in positive colon cancer cells. Collectively, FLU inhibited the proliferation of colon cancer cells by suppressing mitosis and inducing apoptosis and cell senescence [18]. Studies have shown that the interference with microtubules can induce tumor cells’ senescence to inhibit cancer cell growth, which is mediated by microtubule destruction [94]. Whether FLU can induce colon cancer cells’ senescence through its inhibition of microtubule needs to be elucidated.

## 9. Flubendazole Inhibits Oral Squamous Cell Cancer by Suppressing Cell Migration and EMT

Oral squamous cell cancer (OSCC) is one of the most common malignant tumors which affects more than 400,000 people a year. Because of its high recurrence and metastasis rate, OSCC has a poor prognosis and high mortality [95,96,97]. The process of EMT is closely related to the occurrence of cancer [98,99]. Vera Kralova and colleagues found that FLU decreased the viability of the oral squamous cancer cells (PE/CA-PJ15 cells and H376 cells) and the precancerous oral keratinocytes. While oral keratinocytes and normal gingival fibroblasts were not significantly influenced by FLU. FLU inhibited PE/CA-PJ15 cells migration by decreasing the levels of the proteins, which regulates cell migration, including focal adhesion kinase FAK, Rho-A, Rac1 GTPases, and Rho guanine nucleotide exchange factor GEF-H1. FLU inhibited EMT, as well as cadherin switching, induced by TGF-β by reducing the level of N-cadherin in oral keratinocytes DOK cells, indicating that FLU suppressed OSCC by inhibiting cellular migration, and EMT in oral squamous cancer cells [19].

## 10. Conclusions

As a member of benzimidazole anthelmintics, FLU has been reported to have anti-tumor effects. In this review, we summarized as follows:

(1) FLU suppresses breast cancer through the inhibition of proliferation, metastasis and drug resistance mainly by targeting breast CS-like cells via inhibiting cell cycle progression and tubulin polymerization; (2) FLU suppresses TNBC mainly through the inhibition of breast CS-like cells via inhibiting STAT3 activation; (3) FLU mitigates resistance to trastuzumab through the inhibition of CS-like cell properties and the HER2 pathway, and the induction of apoptosis and G2/M phase arrest in HER2-positive breast cancer; (4) FLU inhibits TNBC by promoting autophagic cell death of TNBC cells by promoting EVA1A; (5) FLU suppresses melanoma by mitosis inhibition and apoptosis induction in melanoma cells; (6) FLU inhibits the growth and metastasis of melanoma through silencing of the immunosuppressive effects of PD-1 and MDSC; (7) FLU inhibits CRPC growth through the promotion of the p53 signaling pathway to induce ferroptosis and cell cycle arrest; (8) FLU inhibits the proliferation of NSCLC cells by the induction of autophagic cell death (needs to be further elucidated); (9) FLU suppresses the proliferation of colon cancer cells through the inhibition of mitosis, and induction of apoptosis and cell senescence; (10) FLU suppresses OSCC through the inhibition of cellular migration, and EMT in oral squamous cancer cells (Table 1). It can be seen from the above that the mechanism of FLU inhibiting tumors is to: (1) inhibit cell microtubules, resulting in cell cycle disorder; (2) induce apoptosis; (3) silence the immunosuppressive effects of PD-1 and MDSC; (4) promote tumor cell senescence. These four anti-tumor mechanisms of FLU need to be further studied. FLU mainly plays a role in binding and inhibiting microtubules, so whether the other three anti-tumor mechanisms of FLU are related to its inhibition of microtubules needs to be further explored. Moreover, other anti-tumor mechanisms of FLU, such as glucose transport disorder, need to be further studied. The side effects of FLU in anti-tumor, such as promoting aging, also need to be clarified. It can be seen from the above that FLU can play an anti-tumor role through the p53 and STAT3 signaling pathways. Whether there are other signal pathways involved in the anti-tumor effect of FLU remains to be further studied. Furthermore, it can be seen from the above that FLU plays an anti-tumor role by inducing EVA1A-mediated autophagic cell death, therefore, the role of EVA1A in the anti-tumor process of FLU is worth studying.

It is believed that with in-depth studies of FLU and its anti-tumor effect, FLU will become a new anti-tumor drug.

## Figures and Tables

**Table 1:** Summary of antitumor effects of flubendazole in different tumors.

Type of Cancer	Antitumor Mechanism of Flubendazole	Experimental Model	Reference
CS-like cells-enriched breast cancer	Inhibition of breast CS-like cells via suppressing cellcycle progression and tubulin polymerization in breast cancer cells	Breast cancer cell(MDA-MB-231, BT-549, MCF-7 and SK-BR-3 cells) and mouse allograft breast cancer model	[24]
Triple-negative breast cancers (TNBC)	Inhibition of proliferation and metastasis maily by suppressing breast CS-like cells via suppressing STAT3 activation	TNBC cell lines: (MDA-MB-231,Hs578T, BT-549 and 4T1-Luc) and mouse allograft breast cancer model	[23]
Human epidermal growth factor receptor-2 (HER2-positive breast cancer)	Improvement in drug resistance through suppressing CS-like cells properties and HER2 pathway, and inducing apoptosis and G2/M phase arrest	Human breast cancer cell lines (BT474,SKBR3, MDA-MB-453) and mouse allograft breast cancer model	[22]
Triple-negative breast cancers (TNBC)	Promotion of autophagic cell death of TNBC cells by promoting EVA1A	TNBC cell lines and mouse allograft breast cancer model	[70]
Melanoma	Inhibition of mitosis and induction of apoptosis	Human melanoma cell lines	[14]
Melanoma	Silencing of immunosuppressive effects of PD-1 and MDSC	Melanoma cell line (MDA-MB-435) and mouse xenograft melanoma model	[20]
Castration-resistant human prostate cancer (CRPC)	Promotion of p53 signaling pathway to induce ferroptosis and cell cycle arrest	human CRPC cell lines (PC3, DU145)	[87]
Non-small cell lung cancer (NSCLC)	Induction of autophagic cell death (needs to be further elucidated)	NSCLC cell lines (A549, H460)	[27]
Colon cancer	Inhibition of mitosis and induction of apoptosis and cells senescence	Human colon cell lines (SW480 and SW620)	[18]
Oral squamous cell cancer(OSCC)	Inhibition of cell migration and epithelial–mesenchymal transition (EMT)	oral squamous cancer cells (PE/CA-PJ15 cells and H376 cells) and precancerous oral keratinocytes	[19]
